# Water Chreodes and the Mechanisms of Ligand Diffusion, General Anesthesia, and Sleep

**DOI:** 10.1155/2011/396560

**Published:** 2011-07-26

**Authors:** Lemont B. Kier

**Affiliations:** Center for the Study of Biological Complexity, Virginia Commonwealth University, Richmond, VA 842030, USA

## Abstract

The concept of the presence of passageways, chreodes, created by the influence of the hydropathic states of amino acid side chains on the surface water of proteins, has been proposed. These chreodes facilitate and direct the diffusion of neurotransmitters through surface water, to the receptor or active site on a protein. This system of chreodes is vulnerable to the presence of some other molecules that may encounter the chreode system. This encounter and disruption has been proposed to explain the mechanism of general anesthesia. Based on much recent evidence of the similarities between anesthesia from volatile anesthetic agents and sleep, a comparable mechanism has been proposed for sleep. Since this must be an exogenous substance to be comparable to a general anesthetic agent, it was proposed that this exogenous, sleep-producing substance is elemental nitrogen. Recent evidence supports these hypotheses.

## 1. Ligand Diffusion

A fundamental process in all living systems is the movement of molecules toward each other, followed by frequent encounters and changes in their states. Invariably this movement, or diffusion, takes place in water. This movement is influenced by attributes associated with these molecules and other features that they encounter along the way. It is appreciated that a two-dimensional diffusion near the protein surface, relative to a three-dimensional diffusion in bulk water, enhances the rate of movement in this process [1–3]. In the case of a ligand molecule encountering an effector, the approach of the ligand initially comes to the surface of the protein housing the effector. This system is typical of a transmitter-receptor or substrate-enzyme system. Early studies [4] of this process proposed a change in local water viscosity on the protein surface playing a role in the rate of enzymatic reactions. This influence on water viscosity changes was demonstrated using circular dichroism studies [5]. This viscosity change was proposed to create a change in the structure of bulk water on the protein surface leading to a facilitation of the diffusion of a substrate to the enzyme active site.

A model of this process was created [6] in which a ligand approaches a receptor and becomes engaged in a pool or pathway near the receptor. This example focused on the sweet-tasting receptor. Residence of sweet-tasting molecules in this pathway or pool was proposed to explain the phenomenon of persistence of the taste when the system was washed out. This persistence phenomenon is encountered in many ligand-effector systems. The concept provides an argument for a possible diffusion direction influence existing near the protein surface as well as a persistent presence of a ligand near the receptor. These studies lead to the possibility that structural features on the protein surface may influence the water near the surface in a way that guidance and diffusion facilitation of a ligand to an effector is achieved. Reflection on this possibility leads to a conclusion that molecule-molecule interactions in a living system cannot be random events. Consider a vast protein network functioning collectively on a macroscale. The encounters must be focused, occur on a narrow temporal scale, and be highly selective to minimize interfering encounters. 

This complex issue was recently addressed [7]. A proposal was put forward that the surface of a protein, containing a variety of amino acid side chains, was influencing the water in the vicinity of the surface. This influence is due to the hydropathic states of each side chain. The twenty amino acids have a rich variety of this attribute ranging from very hydrophobic to very hydrophilic. See Table 1. A series of models of water in the presence of these side chains showed that this side chain attribute can influence the organization of water in the vicinity. These studies began with the use of an agent-based modeling system known as cellular automata, to model bulk water [8]. A valid model of bulk water was achieved leading to a series of modeling studies dealing with water, solutes, and stationary side chains in bulk water [9–12]. The models revealed a strong influence of side chains on the organized state of nearby bulk water. Interpreting these as side chain characteristics, it was proposed that distinct patterns of side chains on the surface of an active protein may exist [7]. These patterns were proposed to create diffusion influences on the water near the protein surface. Since water is approximately 1/3 void, these spaces may be organized from the variety of hydropathic states present to create a pathway, called a chreode, facilitating diffusion [7]. The consequences of such a chreode are to retain a ligand near the protein surface and to facilitate its diffusion to the effector. If the effector is an enzyme, the encounter with the substrate may be followed by a rapid diffusion of the product away from the enzyme via the presence of a chreode that has evolved to carry out this function. Thus an enzymatic reaction is fast due to rapid approach of the substrate and a rapid departure of the product from the enzyme. 

Several characteristics are postulated to be associated with each chreode near a particular effector. Each chreode is selective for a particular effector on a particular protein, thus the chreodes found on a protein associated with an acetylcholine receptor, will be found on all such proteins and will have some specificity for the acetylcholine using that chreode. The chreode may be viewed as part of the effector with a role of a diffusion facilitator. A chreode is not a fixed pathway on the protein surface. It is an evanescent structure that forms, breaks up, and forms again, but with some regularity it retains a basic organization permitting the diffusion of the ligand to the effector. Finally the chreode associated with a particular effector might play a role in excluding a nonfunctioning molecule with a structure different from that molecule associated with the effector.

Models of the chreode concept showed the effect on the diffusion rates of solutes simulating ligands [13–15]. Other models of the chreode influence on diffusion, revealed that other molecules randomly dispersed among the chreodes and ligands produced a reduction in the diffusion rate [13]. The potential susceptibility of the chreodes to functional alteration led to an hypothesis of the mechanism of action of volatile anesthetic agents [16].

## 2. General Anesthesia

The nonspecific, volatile, general anesthetic agents represented by diethyl ether, nitrous oxide, and the halothanes have been used and studied for many years. Early explanations of their actions focused on their lipophilic character related to the aqueous and fatty tissues in the body [17, 18]. Some proposed mechanisms have recently been summarized [19]. The possibility of a cell membrane being the target for a volatile anesthetic agent has been refuted by evidence that stereoisomers have a small difference in potency [20–22]. The role of membranes as targets would require a far higher concentration than those used clinically [23, 24]. The possibility of a protein specific target has been considered but rejected on the basis of concentrations needed to produce anesthesia [25–27]. The possible involvement of specific receptors has been considered. These include gamma-amino butyric acid [GABA] [20, 28]. Anesthetic agents employed do not produce a response pattern characteristic of direct GABA receptor behavior [29–32]. Other proposals invoke the role of several binding sites such as glutamate and acetylcholine [33, 34]. Other studies illustrate the roles of gases in producing anesthesia [35, 36]. 

Further evidence of multiple binding sites comes from a steep dose-response effect [37]. Tolerance is not experienced with volatile anesthetic agents, an attribute usually experienced with drugs acting at specific receptors. The evidence to date leads to the current view that volatile anesthetic agents produce a series of weak binding events near several different receptors. The response observed transcends any specific receptor and is due to a complex summation of modest effects from several receptors. This concept led to a consideration of the chreodes associated with several different receptor systems as targets of volatile anesthetic agents [7, 16].

## 3. A Chreode Theory of General Anesthesia

The nature of water chreodes, just defined, suggests a possible target for a molecule that can interrupt or interfere with their function. Each volatile anesthetic agent is relatively inert chemically, has a modest size, and is moderately hydrophobic. Each chreode system, hypothesized to exist in support of a receptor, is potentially vulnerable to alteration by some other molecules. This is possible because the chreode existence is postulated to be entirely dependent upon hydrophobic and hydrophilic attributes of the amino acid side chains on the receptor protein, creating it. A theory of this interference with the chreode structure by a volatile anesthetic agent has been proposed [16]. This interference with chreodes leads to a reduction in their function, which is to facilitate and direct transmitter molecule diffusion to its receptor. The variety of these receptor types influenced, and the modest degree of the interference, is compatible with the current understanding of general anesthetic mechanisms [19, 26]. The inspired volatile anesthetic agent diffuses through a large part of the body, encountering several neurotransmitter systems. It interferes with several systems by altering the chreode function in each system to some extent. The sum of these ligand diffusion alterations produces the observed level of general anesthetic response, analgesia, anesthesia, amnesia, and muscle function loss [16]. At the end of the anesthetic administration, there is a decline in the concentration of this agent throughout the body, leading to a recovery of the chreode functions and a recovery of the neurotransmitter systems that they have been influencing. At some point, there is a return to consciousness and a recovery of the systems inhibited in the patient. 

## 4. General Anesthesia and Sleep

There is an emerging awareness of the similarities between general anesthesia and sleep [38–41]. These similarities include physiological and behavioral patterns. Both sleep and anesthesia involve wakefulness mediated in the CNS [38–40]. The pons has been implicated in arousal states [42, 43]. The cholinergic system in the pons has been identified in the arousal state [44, 45]. Sleep is a coordinated response of anesthesia characteristics such as analgesia, unconsciousness, muscle relaxation, and amnesia [46]. General anesthesia interferes with CNS cholinergic systems [23, 47–49], and acetylcholine is more prominent during awake periods than during sleep [44, 45, 50]. In non-REM sleep and anesthesia, the cholinergic system is less active in the pons [51, 52]. The changing of inhaled nitrogen with helium at normal pressures could influence sleep as shown in some studies [53, 54].

These observations of the similarities between anesthesia and sleep led to a hypothesis that a comparable mechanism is operating, centered on neurotransmitters such as acetylcholine, GABA, glutamate, and others. The prime targets are likely in the CNS with peripheral effects certainly operating. It raises the possibility that sleep is produced by an agent outside of the body, capable of interfering with processes that are operational during waking and anesthetic-free periods.

## 5. A Theory of Sleep

The origin of sleep has been proposed, based on the roles of chreodes and the similarities between sleep and anesthesia produced by the volatile anesthetic agents [55]. It was proposed that the basic mechanism is the same, the interference of chreodes associated with several critical neurotransmitters, discussed above. These transmitters are in the CNS and elsewhere where muscle function may be interfered with. An exogenous substance, playing a role comparable to a general anesthetic agent, operates to produce sleep. It was proposed that this exogenous substance is elemental nitrogen, N_2_ [55]. Nitrogen is 78% of the air we breathe. It is ubiquitous in our bodies and along with oxygen it enters and leaves our bodies in a respiratory cycle. 

Nitrogen is chemically inert, not participating in any biochemical or biophysical process identified to date. Nitrogen is a major ingredient taken in with every breath and is largely expelled by the same process. It is proposed that there is a slow accumulation of a small excess of N_2_ in the body over the course of a wakeful period of time. This nitrogen content is distributed everywhere in the body. In some locations, the nitrogen becomes available to interfere with chreodes associated with functional systems. These events mimic the proposed role of a volatile anesthetic agent that interferes with chreodes associated with the function of critical neurotransmitters. In the case of nitrogen, this interference is a much more subtle effect, called sleep. 

During sleep there is a reduction in the respiration rate, dropping to about 75% of the wakeful rate. This rate change produces a reduction in the accumulation of excess nitrogen which leads to a decline in the chreode influence on transmitter diffusion. The outcome of this change is a return to the normal function of the receptors. This is the onset of wakefulness. During the sleep period, there are episodic declines in the effect of nitrogen on the chreodes, producing a shift in the sleep attributes toward wakefulness. These episodes are referred to as rapid eye movements [REM]. As the sleep period progresses, the length of the REM experiences becomes longer until ultimately, the level of nitrogen in the chreodes becomes permanently insufficient to reduce the chreode function, and wakefulness occurs.

It is known that nitrogen will produce a low level of anesthesia but a high pressure must be used [56]. The conclusion of that study was the reinforcement of the concept that an agent, including nitrogen, acting as an anesthetic, is nonspecific in its targets. The potency exhibited by nitrogen in that study was low and so the low level of anesthesia achieved through breathing would account for the characteristics of sleep. Other observations support this hypothesis. It is observed that patients with sleep deprivation require significantly less general anesthetics to produce anesthesia [57]. A study of the effect of nitrogen on ion channel currents revealed an effect identical to that from a general anesthetic agent [58, 59]. It is now recognized that there is no clear evidence of a species that does not sleep [60]. The occurrence of sleep is reported in the fly, *D. melanogaster *[61, 62], zebra fish [63, 64], and the worm *C. elegans* [65].

Evidence is presented in this paper of the existence of chreodes facilitating ligand diffusion over protein surfaces. That diffusion is proposed to be interrupted by general anesthetics to produce anesthesia and by inhaled nitrogen to produce sleep. Each concept bears further evaluation of experimental evidence. Such novel hypotheses present a challenges for further study.

## Figures and Tables

**Table 1:** Hydropathic states of amino acid side chains.

Amino Acid	Hydrophobicity^a^
Arg	−1.01
Lys	−0.99
Asp	−0.77
Glu	−0.64
Asn	−0.60
Gln	−0.22
Ser	−0.04
Gly	0.00
His	0.13
Thr	0.26
Ala	0.31
Tyr	0.96
Val	1.22
Met	1.23
Cys	1.54
Leu	1.70
Phe	1,79
Ile	1,80
Trp	2.25

^a^Pi values calculated: J. Fauchere, M. Charton, L. Kier, A Verloop, and V. Pliska. Amino acid side chain parameters for correlation studies in biology and pharmacology. *Int. J. Protein Res.* **32, **269–278 [1988]. The negative values are hydrophilic; the positive values are hydrophobic.
